# Length Effect at Testing Splitting Tensile Strength of Concrete

**DOI:** 10.3390/ma15010250

**Published:** 2021-12-29

**Authors:** Marta Słowik, Amanda Akram

**Affiliations:** Department of Structural Engineering, Faculty of Civil Engineering and Architecture, Lublin University of Technology, Nadbystrzycka 40, 20-618 Lublin, Poland; a.akram@pollub.pl

**Keywords:** concrete, tensile strength, length effect, size effect

## Abstract

Tensile strength of concrete is the basic property when estimating the cracking resistance of the structure and when analysing fracture processes in concrete. The most common way of testing tensile strength is the Brazilian method. It has been noticed that the shape and size of specimens influence the tensile splitting strength. The experiments were performed to investigate the impact of cylinder’s length on tensile concrete strength received in the Brazilian method. During the experiment the tensile concrete strength was tested on two different sizes cylindrical specimens: 150 mm × 150 mm and 150 mm × 300 mm. Experiments were performed in two stages, with two types of maximum aggregate size: 16 mm and 22 mm. The software “Statistica” was used to perform the broad scale statistical analysis. When comparing test results for shorter and longer specimens, the increase of tensile splitting strength tested on shorter cylinders was observed (approximately 5%). However, when performing deeper statistical analysis, it has been found that the length effect was not sensitive to the strength of the cement matrix and the type of aggregate but was influenced by the aggregate size. Further experiments are needed in order to perform a multi-parameter statistical analysis of scale effect when testing the splitting tensile strength of concrete.

## 1. Introduction

Concrete is considered as a quasi-brittle material. Tensile strength of concrete is much lower than its compressive strength. Although the compressive strength of concrete is of paramount importance when designing concrete structures, the tensile concrete strength cannot be neglected in design. The tensile concrete strength is the basic property when estimating cracking resistance of structure and when analysing fracture processes in concrete. Furthermore, the tensile strength plays the important role not only in case of serviceability states but also in some cases of ultimate states, for example flexural capacity of unreinforced and slightly reinforced members, shear capacity in members without shear reinforcement, bond failure and minimum reinforcement. Some aspects of the utilization of concrete tensile strength in the design of concrete structures have been discussed in the CEB Bulletin No. 237 [1].

Concrete is a composite material. The bulk of concrete is made up of fine and coarse aggregates (crushed or gravel) and therefore concrete properties are significantly influenced by the aggregate type and granulation [2,3,4]. However, the hydrated cement matrix also affects concrete strength [5,6]. It should be pointed that the internal structure of hardened concrete consists of aggregate grains and cement matrix as well as the initial system of defects like microcracks, unhydrated cement grains, voids and pores. Furthermore, the internal defects depend not only on the material composition but also on the ambient conditions like humidity and temperature, restrains which can influence changes in concrete internal structure and concrete properties over time [7]. The existence of weaker and stronger regions in the internal structure of concrete makes the material strongly heterogeneous. In turn, this heterogeneity is correlated with possible randomness of laboratory results concerning strength parameters or the fracture behaviour of concrete [8].

All these conditions influence concrete quality and necessitate experimental testing of concrete properties and evaluating test results using statistical methods. Tests should be performed at standard conditions and on standardized specimens. When designing concrete structure on the basis of rules given in constructional standards like Eurocode 2 [9], American Standard ACI318 [10] and others, a uniaxial compressive strength and an uniaxial tensile strength should be used. There are not problems when performing the uniaxial compressive test whereas it is much more difficult to realize a stable uniaxial tensile test. Therefore, indirect methods are usually applied to determine the tensile strength of concrete and as a standard method the splitting tensile test is mostly recommended, for example in the European code [11]. The splitting test is called the Brazilian method since it was proposed by Brazilian researchers Carneiro and Barcellos [12]. The test can be performed on cubic or cylindrical specimens of different dimensions.

The modes of concrete failure in compression are subject to a size effect. In general, smaller specimens exhibits greater stress at failure than larger ones. Some findings reported in the literature suggest that size effect exists also when testing other properties of concrete like tensile strength [13,14,15,16] and fracture energy [17,18]. Unfortunately, this problem is much less recognized and described, probably because test results of tensile strength and fracture energy are characterized by higher deviation than the results of compressive strength. Furthermore, the specimen’s tensile strength is to a larger extent related to the condition of testing. As the Brazilian splitting test is mostly performed on cylindrical specimens, it is important to recognize the effect of the cylinder’s dimensions on tensile strength. In the paper the experimental investigation is presented on how the length of cylindrical specimens influences the tensile splitting strength of concrete.

## 2. Materials and Methods Tensile Strength of Concrete—Test Methods and Size Effect

**Figure 1:**
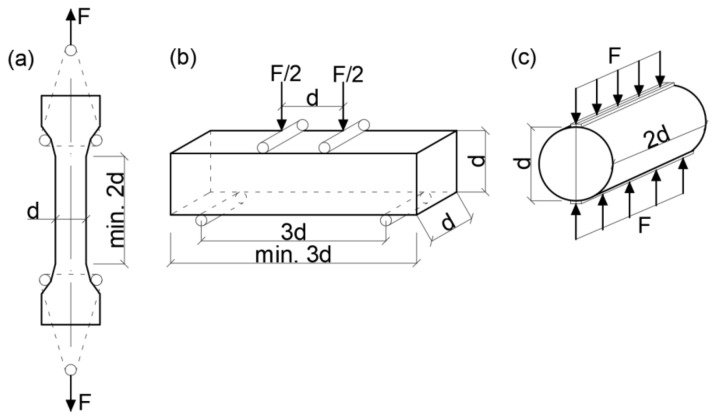
Test methods of tensile strength of concrete: (**a**) uniaxial tensile test, (**b**) flexural test, (**c**) split cylinder test.

The basic methodology to obtain concrete tensile strength should be a uniaxial tensile test. The proper assessment of the uniaxial tensile strength of concrete is considerably affected by the testing technique. The specimen configuration and the local stress concentration, the type of the gripping system and the testing machine stiffness are important factors which affect the obtained results [7]. Even when the test is carefully executed, the test results may be influenced by some concrete eccentricities due to the heterogeneity of internal structure which is caused, for example, by an irregular distribution of aggregate grains. The necessity of a highly precise force application and the necessity of ensuring static loading make the performance of the uniaxial test too difficult to complete in several laboratories. Therefore, the tensile strength of concrete is usually determined using indirect methods. Indirect techniques of tensile strength measuring which are the most frequently used are: the split cylinder test [11] and the flexural test [19]. It should be noted that testing tensile strength by indirect methods, both the flexural and the splitting one gives higher value of tensile strength than the strength obtained in the uniaxial test. Well-known test methods of measuring tensile strength of concrete are shown in the Figure 1. However, research is still looking for simple and adequate test methods which could be applied in practice. The new method of testing tensile strength of concrete has been recently proposed by Resan et al. [20] and Liao et al. [21].

**Figure 2:**
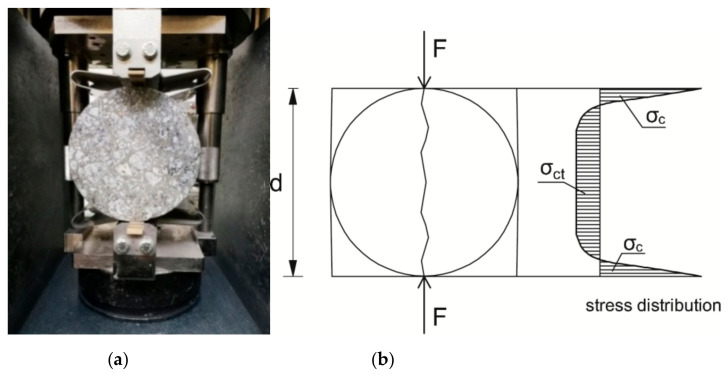
The Brazilian splitting test: specimen in the test stand (**a**), stress distribution in cross section σ_ct_—tensile stress, σ_c_—compressive stress (**b**).

The uniaxial tensile test is conducted on rectangular prisms of a cross section 100 mm × 100 mm or 150 mm × 150 mm and the length equals two times the specimen’s width. The flexural tensile test can be performed using a beam specimen in three point bending test and four point bending test. The tensile strength obtained in the three-point bending test is 13% higher compared to the strength from the four point bending test. Therefore, the loading arrangement by two concentrated forces is recommended in the code [19]. In the Brazilian splitting test, cylindrical or cubic specimens can be used. A specimen is placed in the compression testing machine (cylinders in horizontal position) and the load is applied through plywood strips situated under and over the specimen in central position. Although the specimen is compressed but in the predominant depth of cross section a tensile stress is generated by the Poisson’s effect and this makes the specimen split in tension into two pieces (almost halves) as it is presented in Figure 2. The methods of testing tensile strength of concrete are described at length in [22].

The concrete splitting tensile strength is calculated from Equation (1).

(1) $$f_{\mathrm{ct},\mathrm{sp}}=\frac{2\cdot F}{\pi \cdot L\cdot d},$$

where: F—failure load, L—specimen’s length, d—dimension of a specimen’s cross section.

When designing concrete structures, the uniaxial tensile stress should be used. Noting that the values obtained of the Brazilian splitting method are higher than those got from the uniaxial tensile test, the conversion factor is recommended in Eurocode 2 [9], (Equation (2)).

(2) $$f_{\mathrm{ct}}=0.9f_{\mathrm{ct},\mathrm{sp}},$$

**Figure 3:**
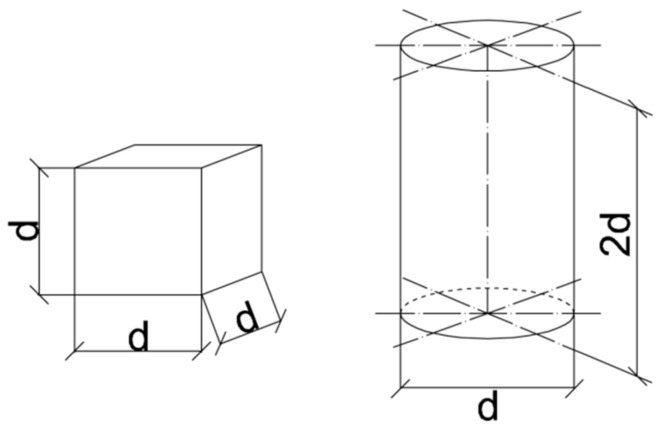
Types of specimens for testing tensile strength of concrete. Nominal dimensions for cubes: d = 100, 150, 200, 250, 300 mm, and for cylinders: d = 100, 113, 150, 200, 250, 300 mm, with acceptable dimension discrepancy ± 10%.

Specimens which are admitted for testing splitting tensile strength of concrete are presented in Figure 3.

It has been noticed that the shape and size of specimens influence the tensile strength of concrete [23,24]. There are some experimental evidence on the existence of the shape effect when testing tensile concrete strength. The tensile strength obtained on cubes exhibits approximately 10% higher values than on cylinders. Such comment can be found in the European code [11]. Therefore, cylindrical specimens are mostly used when performing the Brazilian test. The second important experimental problem is the size of tested specimens. As the cylinder 150 mm in diameter and 300 mm long is the recommended specimen in the European code [25] it is mostly applied in practice, all the more such type of specimen can be also used when testing compressive strength of concrete and modulus of elasticity.

**Figure 4:**
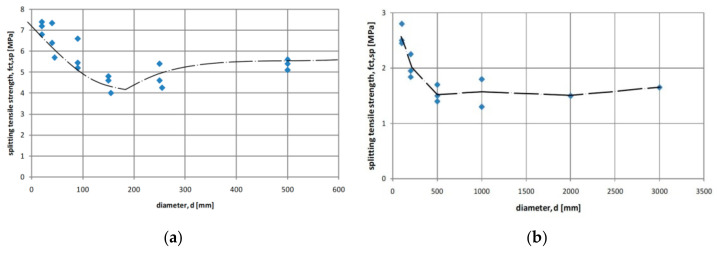
The influence of the cylinder’s diameter on splitting tensile strength. Test results based on Hasegawa et al. [26] (**a**) and based on Bažant et al. [27] (**b**).

The size effect in tension has not been recognized clearly although some efforts were devoted to this matter. Interesting experiments on size effect in the Brazilian split cylinder test were presented in [26]. Tests were performed on 23 cylindrical specimens of different diameter from 100 mm to 3000 mm. All specimens had the length l = 500 mm. The clear experimental evidence for a size effect was noticed when changing diameter from 100 to 500 mm. A further increase in diameter did not influence tensile strength. The obtained results are presented in Figure 4a. In experiments performed by Bažant et al. [27] the decrease of tensile strength was noticed in the specimens of diameter up to 200 mm followed by a plateau or even a slight increase of tensile strength with increasing the diameter up to 600 mm, as it is illustrated in Figure 4b.

Although the effect of specimen’s diameter on the tensile strength have been reported, however the unique trend cannot be deduce from the available data. The main reason of this lays in a high scatter of test results but the fact that the splitting tensile test is sensitive to the boundary conditions, for example the type and the width of strips [28] and the loading rate, is also of importance. When analysing size effect in testing tensile strength, only the diameter of cylindrical specimens has been considered by researchers. The question arises on how the cylinder’s length affects the tensile strength of concrete. The experiments were performed to investigate this problem.

## 3. Experimental Investigation

The experimental investigation was performed to analyse the influence of a cylinder’s length on the concrete splitting tensile strength. During the experiment the tensile concrete strength was tested on two different sizes cylindrical specimens. Cylinders were characterized by the same diameter 150 mm and varied different length 300 mm and 150 mm. Six concrete mixtures were designed for forming the specimens. The additional variable parameters during the experiment were: two types of aggregate (natural gravel from deposits in Poland and crushed granite from deposits in Poland), two maximum sizes of aggregate (16 mm and 22 mm), and two cement-water ratios (C/W = 1.8 and C/W = 2.6). Taking into account the changing parameters of the mixtures, the following symbols for series of tested specimens were applied:

- H16N (higher strength concrete with maximum natural aggregate size 16 mm),
- L16N (lower strength concrete with maximum natural aggregate size 16 mm),
- H22N (higher strength concrete with maximum natural aggregate size 22 mm),
- L22N (lower strength concrete with maximum natural aggregate size 22 mm),
- H16G (higher strength concrete with maximum granite aggregate size 16 mm),
- L16G (lower strength concrete with maximum granite aggregate size 16 mm).

The main aim of the experiment was to evaluate the impact of cylinder’s length on tensile concrete strength received in the Brazilian method. When changing other parameters of concrete mixtures, it was also possible to analyse the influence of the concrete matrix strength and the type of aggregate on tensile strength when testing specimens of different length.

The experiments were performed in two stages. In the first stage concrete mixtures were based on the gravel and the granite aggregate, both with the maximum size 16 mm and in the second stage the gravel aggregate of the maximum size 22 mm was used. The compositions of concrete mixtures are shown in Table 1.

The composition of the aggregate grading is the important task when designing concrete mixtures. In the performed experiment, the proportion of aggregate granulation was very similar in all concrete mixtures, both with gravel and granite aggregate and the grading curves were located between the upper and the lower limit curve.

**Figure 5:**
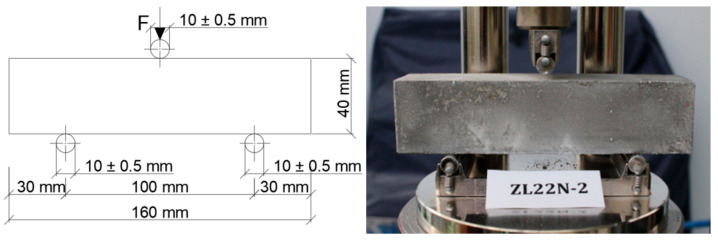
A loading arrangement for testing flexural strength of cement mortar according to EN 196-1 [28].

The strength properties of cement matrix and aggregates were evaluated in additional tests. The strength of hardened cement mortar was tested on beams 40 mm × 40 mm × 160 mm in size. The tests were carried out in accordance with the code [29] in a three-point bending test (in the Automatic Cement Compression and Flexural Tester CONTROLS, model 65-L1300, CONTROLS S.p.A., Milano, Italy), as it is presented in Figure 5. Two types of cement matrices, independently of the aggregate type, were used in concretes during both stages of the experiment. The flexural strength of the stronger matrix (C/W = 2.6) was 8.79 MPa and the flexural strength of the weaker matrix (C/W = 1.8) was 6.86 MPa.

The strength of aggregate was determined on the basis of the aggregate crushing test according to the Polish recommendations [30]. Standardized samples of dried aggregate divided in fractions 4.0–8.0 mm, 8.0–16.0 mm and 16.0–22.0 mm were compressed in a cylindrical mould and then sieved. The aggregate strength was described by the aggregate crushing value which was characterized by the loss of aggregate mass. The results of the crushing test are shown in Table 2.

Concrete compressive strength was tested in the uniaxial compression test on cylindrical specimens 150 mm × 300 mm, according to the procedure given in the code [31]. Strength concrete classes for the series were determined in accordance with [32]. They are presented in Table 3.

The tensile splitting strength of concrete was tested in the Brazilian method (Controls Testing Machine, model 50-C0050/CAL, CONTROLS S.p.A., Milano, Italy). Two types of cylinders were used during the test: the cylindrical specimens 150 mm in diameter and 300 mm in length, and the cylindrical specimen 150 mm in diameter and 150 mm in length. The obtained test results are presented in Table 4. In order to analyse the influence of specimen’s length on tensile strength, the increase/decrease of tensile strength obtained in case of shorter cylinders (150 mm long) compared to the strength tested on typical cylinders 300 mm long was calculated by the formula (Equation (3)):

(3) $$\Delta f_{\mathrm{ct},\mathrm{sp}}=\frac{f_{\mathrm{ct},\mathrm{sp}15/15\;}-{\;f}_{\mathrm{ct},\mathrm{sp}15/30}}{f_{\mathrm{ct},\mathrm{sp}15/30}}\times 100\%,$$

in which: f_ct,sp15/30_—tensile splitting strength tested on cylinder 300 mm long, f_ct,sp15/15_—tensile splitting strength tested on cylinder 150 mm long.

A higher splitting tensile strength of concrete was noted for specimens with nominal dimensions of 150 mm × 150 mm compared to specimens 150 mm × 300 mm for concretes contained natural gravel and crushed granite with the maximum aggregate size 16 mm which were tested in the first stage of experiment. The increase of tensile strength for concretes with the gravel aggregate was 6.6% for the stronger concrete (C/W = 2.6) and 15.9% for the weaker gravel concrete (C/W = 1.8). In case of concretes with the granite aggregate, the strength increase was 2.9% for the lower C/W and 11.7% for the higher C/W. However, this tendency was not confirmed for specimens tested in the second stage of the experiment which were made of concrete with the gravel aggregate of the maximum size 22 mm. In case of those specimens the splitting tensile strength for shorter cylinders were lower compared to longer cylinders. The tensile strength decrease was 4% and 7.5% for stronger concrete (C/W = 2.6) and weaker concrete (C/W = 1.8), respectively.

When analysing tensile strength of concrete together with other changing parameters—the strength of cement matrix and the aggregate crushing (see Table 5), the following conclusions can be drown:

- higher tensile strength was obtained for concretes with granite aggregate, although the lower aggregate strength of gravel mix was measured (higher aggregate crushing value informs of lower aggregate strength); it can be concluded that the shape of aggregate grains (gravel or crushed) is of importance,
- the influence of maximum aggregate size in concretes with gravel aggregate was not clearly observed,
- the increase of the cement matrix strength caused the increase of the tensile strength.

These conclusions were suspected but when investigating length effect, the unique trend cannot be deduced from the obtained test results. It can be concluded that strength properties of aggregate and cement matrix are not significant when investigating the impact of the specimen’s length on the tensile strength of concrete. When analysing the influence of the maximum aggregate size on the tensile strength it was difficult to find coherent conclusions. Therefore, more advanced statistical analysis of test results was performed.

**Table 5:** Increase/decrease of the tensile splitting strength of concrete and strength properties of cement matrix and aggregate.

Stage	Series	Aggregate	Aggregate Crushing–Mean Value [%]	C/W	Flexural Strength of Cement Matrix–Mean Value [MPa]	Increase/Decrease of the Tensile Strength of Concrete Δf_ct,sp_ [%]
I	H16N	gravel 2.0–16.0	10.85	2.6	8.79	+6.6
	L16N	gravel 2.0–16.0	10.85	1.8	6.86	+15.9
	H16G	granite 2.0–16.0	17.79	2.6	8.79	+11.7
	L16G	granite 2.0–16.0	17.79	1.8	6.86	+2.9
II	H22N	gravel 2.0–22.0	10.85	2.6	8.79	−4.0
	L22N	gravel 2.0–22.0	10.85	1.8	6.86	−7.5

## 4. Statistical Analysis

**Figure 6:**
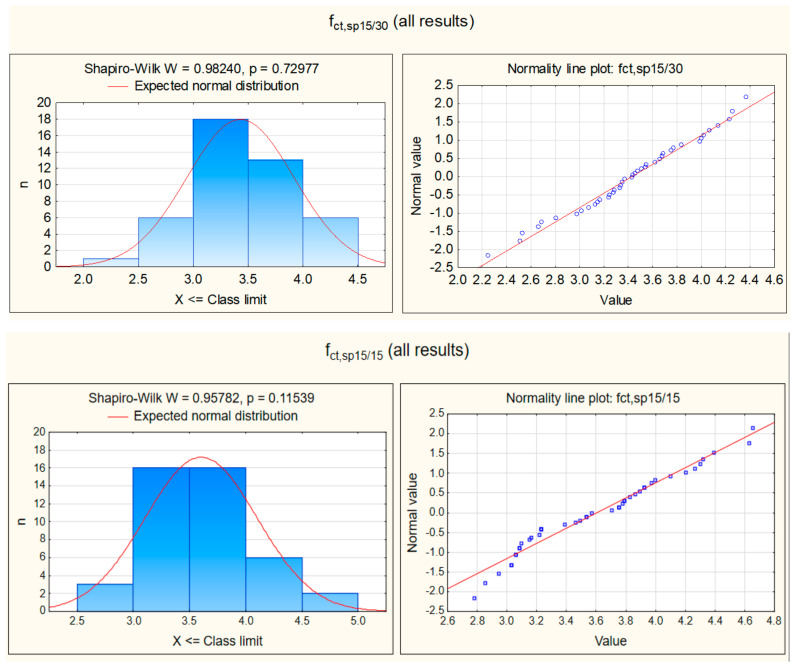
Statistical characteristics of normal distribution for all series taken together, with the results of Shapiro-Wilk test.

The software Statsoft’s “Statistica” (version 13, StatSoft Polska Sp. z o.o, Krakow, Poland) was used to perform the broad scale statistical analysis of the length effect on testing tensile splitting strength of concrete. In the first step of the analysis, the normality Shapiro-Wilk test of the obtained tensile strength results was performed. Two samples were analysed separately: the first one consisted of all test results for longer cylinders and the second one for all test results for shorter cylinders (Figure 6). The normal distribution of the data from both samples was confirmed (w > w_critical_).

**Figure 7:**
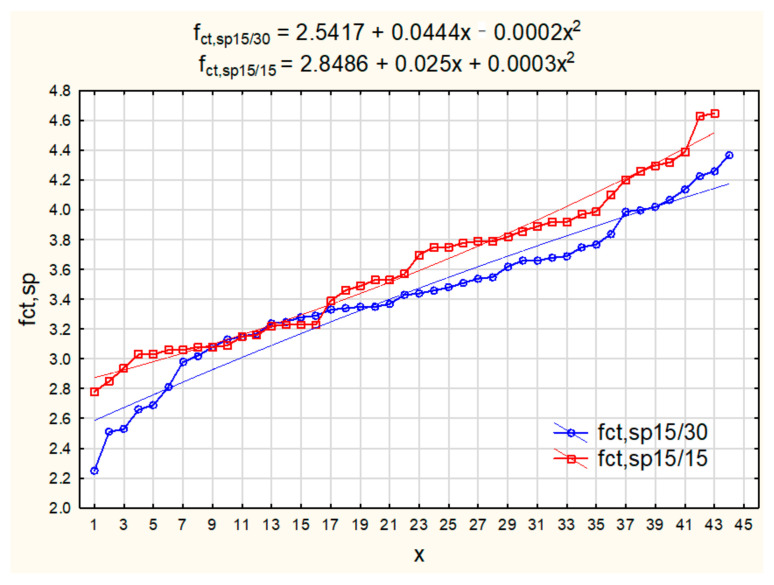
Line plots of variables f_ct,sp15/30_ and f_ct15/15_ for all test results, with the regression functions.

Two regression functions were generated (Equations (4) and (5)), as shown in Figure 7. The regression function for tensile strength of experimental results for short cylinders:

(4) $$f_{\mathrm{ct},\mathrm{sp}15/15}=2.8486+0.025x+0.0003x^{2},$$

The regression function for tensile strength of experimental results for longer cylinders:

(5) $$f_{\mathrm{ct},\mathrm{sp}15/30}=2.5417+0.0444x-0.0002x^{2}$$

The Mean Absolute Percentage Error (MAPE) was calculated in order to examine the fit between the plotted functions and the test results, from the following formula (Equation (6)):

(6) $$\mathrm{MAPE}=\frac{1}{T-n}\overset{T}{\underset{i=T-n}{\sum }}\frac{\left|Y_{i}-Y_{\mathrm{ip}}\right|}{Y_{i}}$$

in which:

T—total number of calculation and forecast periods,
n—number of forecast periods,
Y_i_—the actual value of the variable in the period i,
Y_ip_—the predicted value of the variable in the period i.

The obtained MAPE was 2.8% for tensile strength of longer cylinders and 1.4% for tensile strength of short cylinders. It has been concluded that a good fit was obtained between the generated regression functions and the test data in both cases. The regression functions for longer and shorter cylinders described by Equations (4) and (5) were used to investigate the length effect. The Mean Percentage Error (MPE) was calculated (Equation (7)) in order to analyse the relation between predicted values of the variables f_ct15/15_ and f_ct15/30_.

(7) $$\mathrm{MPE}=\frac{1}{T-n}\overset{T}{\underset{i=T-n}{\sum }}\frac{Y_{i}-Y_{\mathrm{ip}}}{Y_{i}}$$

in which:

T—total number of calculation and forecast periods,
n—number of forecast periods,
Y_i_—the predicted value of the variable fct15/15 in the period *i,*
Y_ip_—the predicted value of the variable fct15/30 in the period *i*.

The MPE between the regression functions generated for longer and shorter specimens was +5.53%. Therefore, it can be concluded that approximately 5% higher tensile splitting strength is obtained when testing concrete strength on cylindrical specimens 150 mm long comparing to tensile splitting strength obtained on cylinders 300 mm long.

The role of other parameters at analysing length effect was examined. It appeared that the flexural strength of cement matrix and the aggregate crushing were not important whereas the maximum aggregate was found to be the significant parameter which also influenced the length effect. Therefore, this parameter was taken into account in the next step of the statistical analysis.

All test results were separated into two groups in accordance with the maximum aggregate size D_max_ = 16 mm and D_max_ = 22 mm. Two samples of data containing test results for longer and shorter cylinders were created in every group. Therefore, four samples were analysed:

- test results of tensile strength tested on cylinder 300 mm long for concretes with D_max_ = 16 mm (series H16N, L16N, H16G and L16G),
- test results of tensile strength tested on cylinder 150 mm long for concretes with D_max_ = 16 mm (series H16N, L16N, H16G and L16G),
- test results of tensile strength tested on cylinder 300 mm long for concretes with D_max_ = 22 mm (series H22N and L22N),
- test results of tensile strength tested on cylinder 150 mm long for concretes with D_max_ = 22 mm (series H22N and L22N).

The Shapiro-Wilk test was applied to investigate the normality of the data distribution. The normal distribution was confirmed for all samples (Table 6).

The regression functions were generated (Equations (8)–(11)):

- for tensile strength values of shorter cylinders, D_max_ = 16 mm:

(8) $$f_{\mathrm{ct}15/15}=2.8291+0.0472x+0.0002x^{2}$$

- for tensile strength values of longer cylinders, D_max_ = 16 mm:

(9) $$f_{\mathrm{ct}15/30}=2.3942+0.0713x-0.0005x^{2}$$

- for tensile strength values of shorter cylinders, D_max_ = 22 mm:

(10) $$f_{\mathrm{ct}15/15}=2.9748-0.016x+0.0106x^{2}$$

- for tensile strength values of longer cylinders, D_max_ = 22 mm:

(11) $$f_{\mathrm{ct}15/15}=3.1682+0.0212x+0.0063x^{2}$$

**Figure 8:**
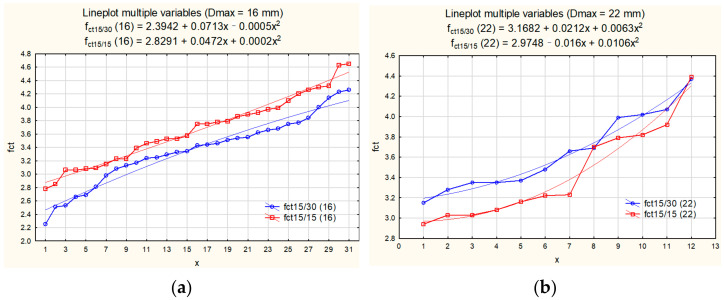
Line plots of variables f_ct,sp15/30_ and f_ct15/15_ with the regression functions: (**a**) for series with D_max_ = 16 mm, (**b**) for series with D_max_ = 22 mm.

The comparison of regression functions for two groups of samples is presented in Figure 8.

The mean absolute percentage error MAPE was calculated from Equation (7) to investigate the generated functions:

- MAPE = 1.41%, for tensile strength values of shorter cylinders, D_max_ = 16 mm,
- MAPE = 2.58%, for tensile strength values of longer cylinders, D_max_ = 16 mm,
- MAPE = 1.96%, for tensile strength values of shorter cylinders, D_max_ = 22 mm,
- MAPE = 1.38%. for tensile strength values of longer cylinders, D_max_ = 22 mm.

Low values of MAPE were obtained and therefore the functions were recognized as a well-fitted approximation of experimental results. The Equations (8)–(11) were applied in the comparative analysis. When comparing regression functions generated for shorter and longer cylinders, the mean percentage error MPE was calculated from Equation (7) in case of two separate statistical samples:

- MPE = + 8.74%, for tensile strength tested on cylinders made of concrete with aggregate D_max_ = 16 mm,
- MPE = −6.21%, for tensile strength tested on cylinders made of concrete with aggregate D_max_ = 22 mm.

When comparing line plots presented in Figure 8a,b, and when analysing the sign of the mean percentage errors (“+” in case D_max_ = 16 mm and “–“ in case of D_max_ = 22 mm), a very interesting and unsuspected observation can be made.

In case of tensile splitting strength of concrete with aggregate of D_max_ = 16 mm (series H16N, L16N, H16G and L16G), the higher strength was obtain when testing tensile strength on cylinders 150 mm long. The length effect has appeared very similar as in the case when investigating all test results together, although a little bit higher MPE was calculated when analysing the limited test data (without test results of concrete with D_max_ = 22 mm). On the contrary, the opposite relation occurred when separating test results for specimens made of concrete with the aggregate D_max_ = 22 mm (series H22N and L22N). The higher tensile splitting test was obtained for cylinders 300 mm long. All test results for shorter cylinders did not exceed the results for long cylinders and it found reflection in a minus sign of the calculated MPE.

These findings indicate that all changing parameters should be taken into consideration and should be examined during the statistical analysis of test data. In the performed research, the maximum aggregate size appeared to be the additional parameter which also played the significant role when analysing length effect at testing tensile splitting strength.

Recapitulating, the effect of the specimen’s length on the tensile splitting strength was observed. However, the performed investigation did not allow to determine a general quantitative relation between tensile splitting strength tested on cylinder 150 mm long and on cylinder 300 mm long, mainly because different conclusions were drown when analysing test data separately for concrete specimens with different maximum size D_max_ = 16 mm and D_max_ = 22 mm.

## 5. Conclusions

Concrete, like other composite materials, consists of fractions with different stiffness. Given that normal strength concrete is a material made up of stiff aggregate particles embedded in a softer hydrated cement matrix, therefore it cannot be used without activating the tensile capacity. Furthermore, compressive failure of concrete is, in reality, a tensile failure perpendicular to the direction of the principal compressive stress. Both compressive as well as tensile failure are subject to a size effect where smaller specimens exhibit higher stresses at failure than larger ones. This effect can be clearly observed when testing strength properties of concrete on different size specimens. The size effect is primarily associated with the utilization of the concrete tensile strength. The most important cases when tensile strength is the necessary design parameter are specifically addressed in the CEB Bulletin no. 237 [1], among them bending of unreinforced and slightly reinforced members, and shear of members without transverse reinforcement.

The proper assessment of the uniaxial tensile strength of concrete is of the paramount importance when designing concrete structures and analysing the failure processes in members made of concrete. The existing test data on testing concrete properties reported in the literature (the broad scale overview is presented in [33]) offer evidence of size effect when testing strength properties of concrete but often the conclusions are not very strong because the data exhibit a large statistical scatter.

Tensile strength of concrete is mostly tested in splitting tensile test. Therefore, the designers have to calculate the uniaxial tensile strength using the simple relation given in Eurocode 2 [9], (Equation (2). More investigations are needed to find a proper relation between uniaxial tensile strength and splitting tensile strength and the investigation of the influence of specimen’s size on splitting tensile strength is the first paramount problem to be resolved. The experiments and statistical analysis of obtained results were performed, and the target of the research was to find the answer for the question whether the length effect when testing splitting tensile strength exists, and in case of a positive answer, to find a simple quantitative relation between test results obtained on specimens of different lengths. However, it was shown that the problem is much more complicated. When comparing test results for 150 mm and 300 mm long cylindrical specimens, the higher tensile splitting strength was obtained on shorter cylinders (approximately 5%). The finding has confirmed the existence of length effect at testing splitting tensile strength. However, when performing a deeper statistical analysis, it has been noticed that such relation did not fit well when separating test results according to other parameters. It was observed that length effect was not sensitive to the strength of cement matrix and the type of aggregate, but it was influenced by the aggregate size.

The performed experimental investigation has shown that the influence of the length of cylindrical specimens on the tensile splitting strength of concrete is an important experimental topic worth to deal with and allowed to indicate the course for further research. The analysis of length effect requires experiments which should be performed on normal and high strength concretes with different type and granulation of aggregate and on more numerous samples. The advanced multi-parameter statistical analysis of the obtained experimental results would help to evaluate the length effect with taking into account not only the size of the specimen but also other parameters, like the maximum aggregate size.

## Figures and Tables

**Table 1:** Composition of concrete mixtures.

	Series	Aggregate Type	C/W	Cement [kg/m^3^]	Water [kg/m^3^]	Sand 0.0–2.0 [kg/m^3^]	Coarse Aggregate 2.0–16.0 [kg/m^3^]	Coarse Aggregate 2.0–22.0 [kg/m^3^]
1	L16G	Granite	1.8	375.6	206.6	481.0	1300.5	-
2	H16G	Granite	2.6	580.7	223.4	421.6	1140.0	-
3	L16N	Gravel	1.8	349.8	192.4	497.1	1344.0	-
4	H16N	Gravel	2.6	533.6	205.2	445.5	1204.5	-
5	L22N	Gravel	1.8	325.9	179.3	512.0	-	1384.3
6	H22N	Gravel	2.6	565.6	217.5	429.3	-	1160.7

**Table 2:** The crushing values for gravel and granite aggregate.

	Type of Aggregate	Fraction [mm]	Aggregate Crushing, Loss of Mass [%]	Aggregate Crushing—Mean Value [%]
1	Gravel	4.0–8.0	6.45	10.85
2	Gravel	8.0–16.0	10.03	
3	Gravel	16.0–22.0	16.06	
4	Granite	4.0–8.0	14.54	17.79
5	Granite	8.0–16.0	17.05	
6	Granite	16.0–22.0	21.79	

**Table 3:** Compressive strength of concrete and strength class of concrete for all series.

	Series	Number of Specimens [-]	Compressive Strength–Mean Value [MPa]	Standard Deviation [MPa]	Strength Class of Concrete [-]
1	H16N	10	48.1	1.72	C 40/50
2	L16N	9	35.1	2.73	C 30/37
3	H16G	10	53.9	3.69	C 45/55
4	L16G	10	41.0	3.48	C 35/45
5	H22N	7	42.8	3.73	C 35/45
6	L22N	7	37.1	0.87	C 30/37

**Table 4:** Tensile splitting strength of concrete—test results.

Series	Cylinders 150 mm × 300 mm			Cylinders 150 mm × 150 mm			Increase (+)/Decrease (–) of Tensile Strength Δf_ct,sp_[%]
	Number of Specimens [-]	Standard Deviation [MPa]	Tensile Splitting Strength—Mean Value f_ct,sp15/30_ [MPa]	Number of Specimens [-]	Standard Deviation [MPa]	Tensile Splitting Strength—Mean Value f_ct,sp15/15_ [MPa]	
H16N	8	0.22	3.54	8	0.29	3.77	+6.6
L16N	8	0.26	2.68	7	0.22	3.11	+15.9
H16G	8	0.47	3.78	8	0.38	4.22	+11.7
L16G	8	0.14	3.40	8	0.33	3.49	+2.9
H22N	6	0.26	3.97	6	0.37	3.81	−4.0
L22N	6	0.11	3.33	6	0.10	3.08	−7.5

**Table 6:** The results of Shapiro-Wilk test for chosen groups of series.

Series	D_max_ [mm]	Number of Specimens [-]	Type of Cylinder [mm]	w	w_critical_	Normal Distribution
H16N, L16N, H16G and L16G	16	32	150 × 300	0.956	0.930	yes
	16	31	150 × 150	0.973	0.929	yes
H22N and L22N	22	12	150 × 300	0.924	0.859	yes
	22	12	150 × 150	0.879	0.859	yes

## Data Availability

Data is contained within the article.
